# 利用CRISPR/Cas9慢病毒系统构建肺部*EZH2*基因敲除小鼠

**DOI:** 10.3779/j.issn.1009-3419.2018.05.02

**Published:** 2018-05-20

**Authors:** 凡荣 孟, 丹 赵, 清华 周, 喆 刘

**Affiliations:** 1 300052 天津，天津医科大学总医院，天津市肺癌研究所，天津市肺癌转移与肿瘤微环境实验室 Tianjin Key Laboratory of Lung Cancer Metastasis and Tumor Microenviroment, Tianjin Lung Cancer Institute, Tianjin Medical University General Hospital, Tianjin 300052, China; 2 300070天津，天津医科大学 Tianjin Medical University, Tianjin 300070, China

**Keywords:** CRISPR/Cas9系统, EZH2, sgRNA, 基因敲除, CRISPR-Cas9, EZH2, sgRNA, Gene knockout

## Abstract

**背景与目的:**

已有的研究证明CRISPR/Cas9（Clustered Regularly Interspaced Short Palindromic Repeats/CRISPR-associated 9）系统是一种能够在哺乳动物细胞中高效操作的新型基因编辑技术，应用人工设计的向导RNA（single-guide RNA, sgRNA）介导外源表达的Cas9蛋白与靶点DNA特异性结合以实现对基因组DNA的切割，被切割后的基因组DNA通过非同源重组或同源重组的方式进行修复，从而实现基因的敲除、或者外源基因的敲入等目标。本研究的目的是应用CRISPR/Cas9技术构建小鼠肺部*EZH2*基因敲除的动物模型。

**方法:**

针对*EZH2*基因的编码区，设计两个靶向*EZH2*基因Exon3和Exon4的sgRNA，通过慢病毒包装、感染细胞、SURVEYOR assay等一系列体外实验，验证所设计的sgRNA的有效性。应用支气管插管的方式把慢病毒灌注到小鼠肺部，利用免疫组化方法和qRT-PCR进行检测。

**结果:**

NIH-3T3细胞的体外实验结果验证了实验所设计的sgEZH2能够有效地在体外细胞系中介导Cas9切割靶DNA；小鼠支气管插管实验及肺部组织免疫组化和qRT-PCR方法检测*EZH2*基因敲除的效率，发现实验组小鼠肺部组织EZH2表达明显降低。

**结论:**

本研究成功设计了两条能够敲除EZH2功能的sgRNA，并应用CRISPR/Cas9技术成功建立了肺部*EZH2*基因敲除的小鼠模型，为研究EZH2的功能和作用机制提供了有效的动物模型。

## Abstract (en)

**Background and objective:**

It has been proven that CRISPR/Cas9 (Clustered Regularly Interspaced Short Palindromic Repeats/CRISPR-associated 9) system was the modern gene-editing technology through the constitutive expression of nucleases Cas9 in the mammalian, which binds to the specific site in the genome mediated by single-guide RNA (sgRNA) at desired genomic loci. The aim of this study is that the animal model of *EZH2* gene knockout was constructed using CRISPR/Cas9 technology.

**Methods:**

In this study, we designed two single-guide RNAs targeting the Exon3 and Exon4 of *EZH2* gene. Then, their gene-targeting efficiency were detected by SURVEYOR assay. The lentivirus was perfused into the lungs of mice by using a bronchial tube and detected by immunohistochemistry and qRT-PCR.

**Results:**

The experimental results of NIH-3T3 cells verify that the designed sgEZH2 can efficiently effect the cleavage of target DNA by Cas9 *in vitro*. The immunohistochemistry and qRT-PCR results showed that the EZH2 expression in experimental group was significantly decreased in the mouse lung tissue.

**Conclusion:**

The study successfully designed two sgRNA which can play a knock-out EZH2 function. An EZH2 knockout animal model was successfully constructed by CRISPR/Cas9 system, and it will be an effective animal model for studying the functions and mechanisms of EZH2.

CRISPR/Cas系统是迄今为止在细菌和古生物菌中发现的第一个也是唯一一个获得性免疫系统，该系统能够特异性识别并结合噬菌体DNA，通过转录产物crRNA（CRISPR-derived RNA）介导Cas蛋白识别并抵御外源性DNA^[1]^。CRISPR/Cas9系统由crRNA和Cas9蛋白组成，结构简单，仅需构建一对引物。CRISPR由一系列短的高度保守的正向重复序列（repeat）与长度相似的间隔序列（spacer）间隔排列组成^[2]^。Cas基因编码的蛋白质具有与核酸结合的功能以及与核酸酶、聚合酶、解旋酶等结合的活性。基于CRISPR/Cas系统的作用原理，研究人员通过体外人工合成sgRNA，成功实现对特定基因片段的精确剪切，由此开创了一种由构建RNA序列介导Cas蛋白识别并剪辑靶基因的新型基因编辑技术^[3]^。在引导RNA的指导下，Cas9定位于特定DNA序列上，进行DNA双链切割，实现基因组的定向编辑。该基因编辑技术具有更高的编辑效率、更简单的操作、成本低、编辑范围广等优势^[4, 5]^。

EZH2为表观遗传抑制因子PcG家族（polycomb group）的重要成员之一，为表观遗传调控因子多梳抑制复合体2（polycomb repressive complex 2, PRC2）的催化亚基，可通过其组蛋白甲基转移酶活性（histone methyltransferase, HMTase）对组蛋白H3的第27位的赖氨酸进行三甲基化（H3K27me3），抑制靶基因转录，参与调控细胞周期、细胞衰老、细胞分化及癌症等生理或病理过程^[6]^。近年来，在多种肿瘤组织中还可以检测到EZH2过表达或突变，证明其与肿瘤的进展也具有密切的联系，且表达水平和肿瘤的恶性程度和不良预后呈正相关^[7]^。已有多篇文献报道EZH2与肺癌^[8]^、淋巴瘤^[9]^、乳腺癌^[10, 11]^、胃癌^[12, 13]^、卵巢癌^[14-16]^等多种肿瘤的发生发展密切相关，具有促进细胞增殖、肿瘤细胞转移和扩散的恶性表型。本研究利用CRISPR/Cas9技术将肺部*EZH2*基因定点敲除，得到肺部敲除*EZH2*基因的转基因小鼠，为深入研究EZH2在肺部肿瘤发生发展中的作用机制奠定基础^[17-19]^。

## 1 材料与方法

### 1.1 材料

#### 1.1.1 所用小鼠、质粒和感受态细胞

实验中所用小鼠均为C57BL/6品系，饲养于SPF级别动物房中，动物房恒温恒湿，昼夜节律为12 h/12 h。质粒载体为psECC，购于Addgene公司，包含Cre和Cas9位点，以及两个BsmB1酶切位点。实验室常规保存质粒iluc、psPAX2、pMD2.G。感受态细胞为DH5α菌株。细胞株为人胚肾293T细胞，小鼠胚胎成纤维NIH-3T3细胞。

#### 1.1.2 试剂

PCR所用高保真聚合酶Q5购于NEB公司。T4 DNA连接酶、T4 PNK、BsmB1限制性内切酶购于NEB公司。酵母提取物和胰蛋白胨购于OXOID公司。DNA Marker购于Invitrogen公司。质粒小量提取试剂盒和无内毒素质粒大提试剂盒购于天根生化科技有限公司。EZH2组化抗体购于Cell Signaling。PCR纯化试剂盒为PureLink PCR Purification Kit。检测试剂盒为GeneArt Genomic Cleavage Detection Kit。麻药2, 2, 2-Tribromoethanol购于Sigma-Aldrich。琼脂糖电泳DNA胶回收试剂盒Gel Extraction Kit购于康为世纪。染料Gelred购于BIOTIUM。DMEM培养基购于BI公司。

#### 1.1.3 仪器

PCR仪器购于Bio-Rad和PowerCycle公司，Nanodrop 2000分光光度计购于Thermo公司，低温台式离心机和低温落地超速离心机均购于Eppendorf公司，倒置荧光显微镜购于OLYMPUS公司。

#### 1.1.4 寡核苷酸序列合成和测序

实验中sgRNA寡核苷酸序列和扩增靶序列所需引物均由上海生工生物工程股份有限公司合成。实验中所需测序样本送由金唯智生物科技有限公司进行测序。

### 1.2 方法

#### 1.2.1 sgRNA的设计和寡核苷酸链合成

根据实验需要设计两个sgRNA（sgEZH2-1, sgEZH2-2），其所在位置分别在EZH2的第3和第4个外显子上（图 1），与BsmB1酶切后形成的黏性末端互补，3’端添加C。设计两对EZH2的CRISPR寡核苷酸链同时，设计一个对照sgRNA-lacZ。表 1为根据实验需要设计的sgRNA寡核苷酸序列oligo1和oligo2，分别在基因的Exon3和Exon4上，同时包括对照寡核苷酸序列sgRNA-lacZ-oligo。并根据靶位点设计相应的扩增引物，分别是sgRNA-Ezh2-F1：5’-ACTTCTGGTGAGTCACTAGATT-3’和sgRNA-Ezh2-R1：5’-AGGGAGGAAAGCAACTGTGAAT-3’，PCR产物长度是510 bp，切割后产物大小是190 bp、320 bp。sgRNA-Ezh2-F2：5’-CCAGAGTTACAGCATCTGTTCA-3’和sgRNA-Ezh2-R2：5’-TGAAACACATAGGTGGCCATCA-3’，PCR产物长度是540 bp，切割后产物大小是160 bp、380 bp。

**1:**
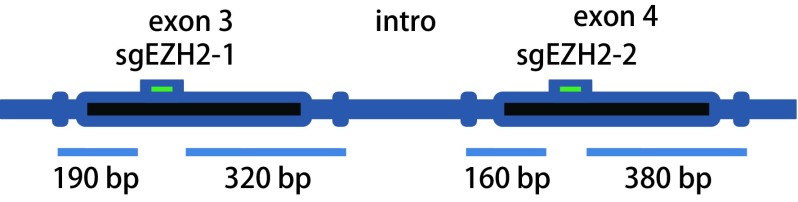
CRISPR/Cas9靶序列在*EZH2*基因的位点及扩增靶序列位点引物示意图 The loci of CRISPR/Cas9 target sequences in *EZH2* gene and the genomic sequences around the Exon3/4-sgRNA-targeting sites

**1:** 构建sgRNA的核苷酸序列 The nucleotide sequence for constructing sgRNA

sgRNA	The nucleotide sequence (5’----3’)
sgRNA-Ezh2-1-oligo1	CACCG CCCTAGTCCCGCGCAATGAGCTC
sgRNA-Ezh2-1-oligo2	AAAC GAGCTCATTGCGCGGGACTAGGG C
sgRNA-Ezh2-2-oligo1	CACCG CCTGAATGCAGTCGCCTCGGTGC
sgRNA-Ezh2-2-oligo2	AAAC GCACCGAGGCGACTGCATTCAGG C
sgRNA-lacZ- oligo1	CACCG CGATCGTAATCACCCGAGTG
sgRNA-lacZ- oligo2	AAAC CACTCGGGTGATTACGATCG C

#### 1.2.2 载体构建

实验所用质粒载体为psECC，自带U6启动子，使用BsmB1限制性内切酶酶切，37 ℃水浴1 h，按照Gel Extraction Kit试剂盒说明书操作回收约12, 017 bp长度的DNA片段。合成的sgRNA的上游（oligo1）和下游（oligo2）引物序列首先配成100 μM工作液，使用T4PNK添加磷酸根，溶液各成分包括：oligo1 1 μL、oligo2 1 μL、10×NEB Buffer 1 μL、T4PNK 0.5 μL，去离子水补体积至10 μL，37 ℃水浴30 min。然后使用PCR仪进行退火程序，形成双链。退火后的sgRNA寡核苷酸双链与回收的psECC载体片段在T4DNA连接酶作用下16 ℃过夜连接，连接产物转化到DH5α感受态中，挑取单克隆菌落并提取质粒p-sgRNA-LacZ、p-sgRNA-Ezh2-1、p-sgRNA-Ezh2-2送测序。

#### 1.2.3 细胞培养和病毒包装

293T细胞用含10%胎牛血清的高糖DMEM培养基于5%CO_2_，37 ℃恒温培养，待细胞密度为大约70%时，质粒经PEI试剂瞬时转染到293T细胞中，此时换为无血清培基。此次病毒包装用三质粒包装系统，包装质粒为pMD2.G和psPAX2，目的质粒为p-sgRNA-LacZ、p-sgRNA-Ezh2-1、p-sgRNA-Ezh2-2。因为实验目的质粒中并无可观察或筛选的标签，无法通过荧光观察或者药物筛选，因此加一个对照目的质粒iluc，此质粒包含绿色荧光蛋白GFP，可观察到绿色荧光，因此便于观察包装系统有无问题。相应的病毒命名为v-iluc、v-sgRNA-LacZ、v-sgRNA-Ezh2-1、v-sgRNA-Ezh2-2（表 2）。293T细胞转染质粒48 h后收集含病毒颗粒的细胞上清，使用低温超速离心机50, 000 g，2 h浓缩，然后弃掉上清，用300 μL PBS复溶，放在4 ℃冰箱过夜，使病毒溶解充分，-80 ℃保存备用，避免反复冻融。

**2:** 慢病毒包装系统 The packaging system of lentivirus

Target plasmids（10 *μ*g）	Virus
iluc	v-iluc
p-sgRNA-LacZ	v-sgRNA-LacZ
p-sgRNA-Ezh2-1	v-sgRNA-Ezh2-1
p-sgRNA-Ezh2-2	v-sgRNA-Ezh2-2
Use 150 mm plate, packing plasmid 1 is psPAX2 (7.5 *μ*g), package plasmid 2 is pMD2. G (5 *μ*g), target plasmid is 10 *μ*g.	

#### 1.2.4 慢病毒感染NIH-3T3细胞及体外敲除效率的验证

于病毒感染前一天取对数生长期的NIH-3T3细胞铺于六孔板中，每孔1×10^5^个，第二天细胞基本长到30%-40%。感染前两小时换为无血清培基，polybrene工作浓度为5 μg/mL，同时加病毒浓缩液各100 μL，孔1加pbs，孔2加病毒v-sgRNA-LacZ，孔3加病毒v-sgRNA-Ezh2-1，孔4加病毒v-sgRNA-Ezh2-2，孔5加病毒v-iluc，每种病毒做三个复孔，混匀，置于5%CO_2_，37 ℃孵箱中培养，6 h后换为无病毒培基。细胞正常培养传代，转至25 cm^2^小培养瓶，培养20天后鉴定。

用胰酶消化六孔板中孔1-5中的细胞，分别取大约1 mL细胞悬液到1.5 mL EP管，细胞个数为5×10^4^-2×10^6^个，200 g，4 ℃离心5 min，弃掉上清，留取细胞沉淀，使用Genomic Cleavage Detection Kit试剂盒进行检测。首先每管加入50 μL细胞溶解液和蛋白消化液的混合液并重悬，重悬液转入PCR管中，PCR仪中程序68 ℃ 15 min，95 ℃ 10 min后温度降为4 ℃。然后吸取重悬液2 μL作为模板，引物为sgRNA-Ezh2-F1R1，sgRNA-Ezh2-F2R2进行PCR程序，95 ℃ 30 s，57 ℃ 30 s，72 ℃ 40 s，40 cycles，其中孔1pbs孔和孔2 v-sgRNA-LacZ孔分别用这两对引物进行扩增，孔3用引物sgRNA-Ezh2-F1R1，孔4用引物sgRNA-Ezh2-F2R2进行PCR片段扩增。两对引物得到的PCR产物长度为510 bp、540 bp。接下来使用PCR产物纯化试剂盒纯化DNA片段，然后进行变性复性，其程序为95 ℃ 5 min，95 ℃-85 ℃-2 ℃/s，85 ℃-25 ℃-0.1 ℃/s。复性之后进行酶切反应，直接在EP管中加入1 μL detection enzyme 37 ℃ 1 h，最后用2%琼脂糖电泳观察条带大小。

#### 1.2.5 通过支气管插管方法把病毒灌注入小鼠肺部

20只实验小鼠共分为四组，每组5只，一组为完全对照组，不做任何处理，一组为对照组灌注病毒v-sgRNA-LacZ，两个实验组灌注病毒v-sgRNA-Ezh2-1和v-sgRNA-Ezh2-2，按照公式体重（g）×400/20来计算每只鼠腹腔注射麻药的量。待麻药发挥作用后，支气管插管方式分别灌入病毒各100 μL，每两周灌注一次，连续四次。小鼠继续饲养8个月后取肺组织，留取标本。提取RNA进行qRT-PCR检测，引物为rtEzh2-F：5’-AGCACAAGTCATCCCGTTAAAG-3’和rtEzh2-R：5’- AATTCTGTTGTAAGGGCGACC-3’；同时对小鼠的肺组织进行石蜡包埋，切片，进行免疫组化分析，观察EZH2基因的敲除效率。

## 2 结果

### 2.1 sgRNA寡核苷酸序列与psECC构建载体

针对*EZH2*基因的Exon3和Exon4分别设计两个sgRNA，并命名为sgEZH2-1和sgEZH2-2，送上海生工生物工程股份有限公司进行寡核苷酸序列的合成。合成后的单链退火形成双链后通过BsmB1酶切位点将其插入psECC质粒载体中，并将构建完成的质粒送金唯智生物科技有限公司测序（图 2），根据测序结果选择序列正确的质粒进行扩增，用于下一步实验。

**2:**
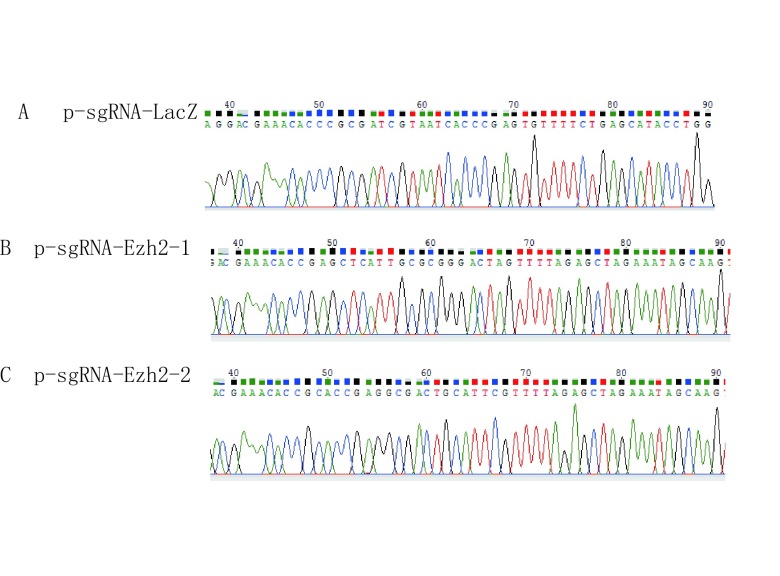
构建质粒测序图 The sequence diagrams of plasmids

### 2.2 构建成功的sgRNA质粒与包装质粒共转染293T细胞包装病毒

正常培养293T细胞至密度为70%-80%，使用PEI作为转染试剂，将目的质粒iluc、p-sgRNA-LacZ、p-sgRNA-Ezh2-1和p-sgRNA-Ezh2-2分别与包装质粒共转染293T细胞，因为实验的目的质粒中并无可观察或筛选的标签，因此质粒iluc作为对照质粒，在荧光显微镜下观察发现，质粒包装体系转入293T细胞后24 h和48 h观察到多数细胞表达较强的绿色荧光（图 3A），说明质粒转染293T细胞及慢病毒包装成功。

**3:**
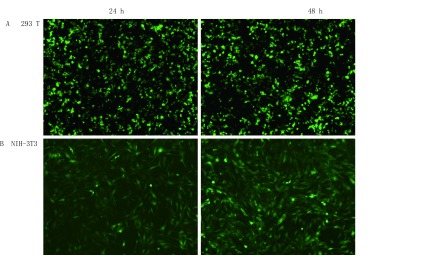
质粒转染293T细胞和病毒感染NIH-3T3细胞。A：293T细胞转染病毒包装质粒和目的质粒iluc后24 h和48 h的荧光强度。B：浓缩慢病毒v-iluc感染细胞NIH-3T3后24 h、48 h的荧光强度。 Plasmid transfected 293T cells and Virus infection NIH-3T3 cells. A: Plasmid transfected 293T cells and the fluorescence intensity of 24 h and 48 h. B: Virus infection NIH-3T3 cells and the fluorescence intensity of 24 h and 48 h.

### 2.3 病毒感染NIH-3T3细胞且鉴定体外CRISPR/Cas9系统敲除*EZH2*基因的效率

浓缩的病毒v-iluc、v-sgRNA-LacZ、v-sgRNA-Ezh2-1和v-sgRNA-Ezh2-2感染细胞NIH-3T3，24h和48h时观察感染v-iluc病毒的细胞荧光强度，大多数细胞显示绿色荧光，说明病毒感染成功（图 3B）。使用v-sgRNA-LacZ、v-sgRNA-Ezh2-1和v-sgRNA-Ezh2-2三种病毒感染的细胞正常培养约20天后，使用Genomic Cleavage Detection Kit试剂盒提取基因组DNA，以其作为模板进行PCR扩增，分别扩增基因组中Exon3和Exon4的部分区域，然后酶切，用2%琼脂糖凝胶进行分析。随后用凝胶定量软件计算切割效率，发现使用病毒v-sgRNA-Ezh2-1和v-sgRNA-Ezh2-2均靶向敲除成功，其切割效率分别为24%和30%（图 4）。

**4:**
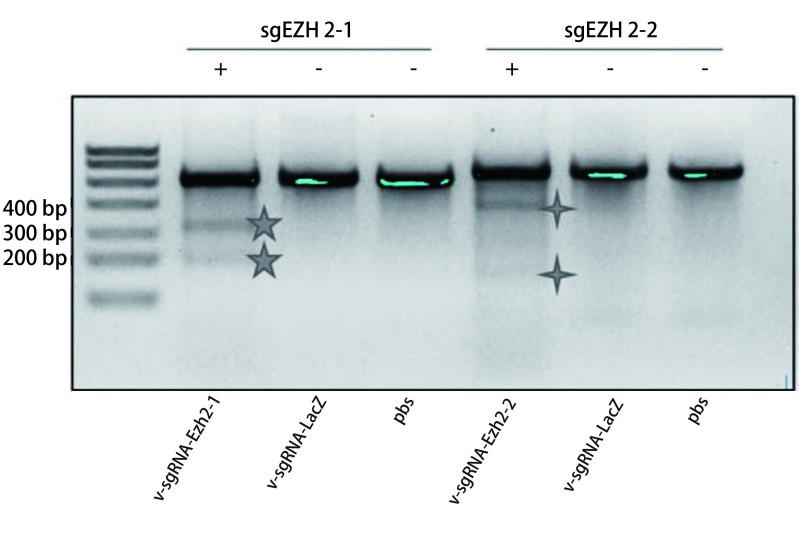
敲除效率验证琼脂糖电泳图。五角星代表sgEZH2-1的切除效率验证，总长度是510 bp，酶切后片段长度是190 bp和320 bp；四角星代表sgEZH2-2的切除效率验证，总长度是540 bp，酶切后片段长度是160 bp和380 bp。其切割效率分别为24%和30%。 Genomic Cleavage Detection Assay Gel. The five-pointed star represents sgEZH2-1 and the total length is 510bp, and the length of the fragment is 190 bp and 320 bp. The four-pointed star represents sgEZH2-2 and The total length is 540 bp, and the length of the fragment is 160 bp and 380 bp. The cutting efficiency is 24% and 30% respectively.

### 2.4 使用支气管插管方法向小鼠肺部灌注病毒，构建小鼠肺部EZH2基因敲除小鼠

为进一步构建肺部EZH2基因敲除小鼠，使用支气管插管方法向小鼠肺部灌注已验证切割效率的浓缩病毒v-sgRNA-Ezh2-1和v-sgRNA-Ezh2-2，小鼠继续饲养约8个月后，留取肺部组织进行石蜡包埋，切片，免疫组织化学方法及qRT-PCR检测，结果显示灌注v-sgRNA-Ezh2-1和v-sgRNA-Ezh2-2病毒液的实验组小鼠EZH2的表达要显著低于空白对照和灌注v-sgRNA-LacZ病毒的小鼠（图 5），以上结果说明已成功构建肺部*EZH2*基因敲除的小鼠模型。

**5:**
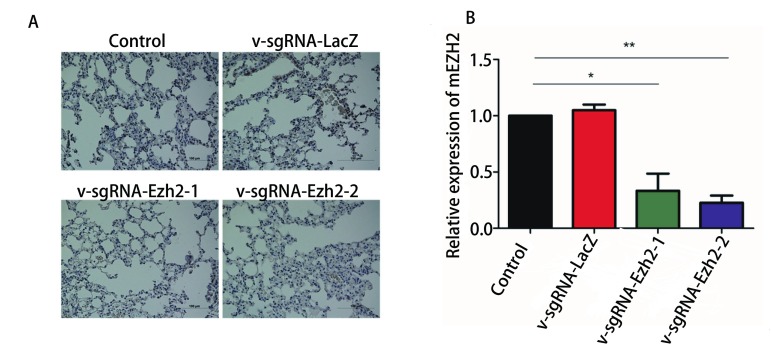
肺部灌注不同慢病毒小鼠的肺部*EZH2*基因敲除结果。A：免疫组化结果；B：qRT-PCR结果；^*^P < 0.05，^**^P < 0.01。 *EZH2* gene knockout results of lung tissue in mice with different lentivirus-infused lungs. A: Immunohistochemistry results; B: qRT-PCR results; ^*^P < 0.05, ^**^P < 0.01.

## 3 讨论

随着生物学研究的发展，基因组编辑技术为了解特定基因功能提供了基础。目前比较常见的锌指核酸内切酶（zinc finger endonuclease, ZFN）和类转录激活因子的效应物核酸酶（transcription activator-like effector nuclease, TALEN）^[20]^技术在很大程度上都被更多样化和高效的CRISPR/Cas9基因编辑系统取代。CRISPR/Cas9系统是一个由蛋白和核酸组成的蛋白核酸复合物，利用sgRNA对特定的DNA序列进行识别，从而介导Cas9蛋白对靶点序列进行切割^[21, 22]^。

本研究应用CRISPR/Cas9技术，以psECC质粒为骨架，快速地构建了用于敲除*EZH2*基因的质粒载体。载体psECC是一个可以同时表达sgRNA、Cas9酶和Cre酶的载体，有四个主要部分：即2.2 kb部分来自LentiCRISPR28的U6-Filler片段，0.3 kb部分来自LentiCRISPR28的EFS启动子，5.3 kb部分Cas9-2A-Cre片段和5.7 kb慢病毒骨架。Sánchez-Rivera等^[23]^使用psECC质粒，在Kras（G12D）驱动的小鼠肺癌模型中，对一系列肺腺癌相关抑癌基因在体内进行了基因编辑实验，以研究这些基因的分子特征，结果表明这种快速体细胞基因组编辑方法能够进行体内基因功能鉴定，显示出psECC载体在体内CRISPR/Cas9应用中的潜力。

在应用CRISPR/Cas9技术时，对于靶位点的选择，主要有两种在线工具：CRISPR Design^[24]^或E-Crispr^[25]^。本研究所应用的sgRNA是应用CRISPR Design设计的。该在线软件针对*EZH2*基因，以NGG作为PAM序列原则，在目标基因序列中选择大约含20个碱基的靶点，并在全基因组序列中扫描潜在的脱靶位点和脱靶效率，经过综合评价，将靶点按照得分高低进行排列，从而选择最合适的靶点。针对EZH2基因选择2个不同靶点，其所在位置分别在EZH2的第3个和第4个外显子上，以确保其中至少1个靶点能实现目标基因的靶向修饰。

将sgRNA克隆至pSECC质粒后，由于质粒片段较大（大约13 kb-14 kb），并不适用于直接转染细胞。因此，本研究应用psECC质粒进行慢病毒包装，以慢病毒感染的方式将目的基因导入细胞。慢病毒感染在基因转染方面有着许多独特的优势：可以感染非分裂期细胞、容纳外源性基因片段大、基因转染效率高、可以长期表达等显著优点。收集浓缩包装psECC后的慢病毒感染细胞NIH-3T3，在荧光显微镜下观察发现，24 h和48 h时90%以上的细胞显示绿色荧光，病毒感染成功。我们的实验结果证明了慢病毒感染体系已成功将编码sgRNA、Cas9蛋白和Cre蛋白的基因导入细胞。

将CRISPR/Cas9基因编辑体系导入细胞后，为确定该体系是否发挥作用，还需要对基因编辑效率进行鉴定。本研究首先应用Genomic Cleavage Detection Kit试剂盒，在体外鉴定CRISPR/Cas9系统敲除*EZH2*基因的效率。该鉴定方法的原理在于，CRISPR/Cas9会对细胞的基因组进行切割，切割后，通过细胞修复机制产生基因组的插入或缺失，通过对基因特异性双链断裂发生的位点进行PCR扩增，PCR产物被变性并重新退火，以致产生错配。随后通过检测酶切割，凝胶电泳和条带密度测定法分析所得条带检测错配比率，进而确定切割的效率，即CRISPR/Cas9的效率。实验结果显示：本研究设计的sgRNA能够有效地在体外细胞系中介导Cas9切割EZH2的DNA，起到敲除EZH2的功能，敲除效率分别为24%和30%。另有研究表明，对细胞进行多次感染，可以增加敲除效率至70%-80% ^[26]^。

将包含敲除*EZH2*基因CRISPR/Cas9系统的浓缩病毒液导入小鼠肺部，采取经口腔气管插管的方式进行。此方式最主要的优点是病毒对气管没有刺激，可以直接抵达目的器官（肺部），利于接种。病毒注入后，小鼠需继续培养大约8个月可见EZH2在肺部的敲除，此种方法可以用来模拟临床上由于*EZH2*基因缺失而诱发肿瘤的过程。肺部组织免疫组化和qRT-PCR方法检测*EZH2*基因敲除的效率，发现实验组小鼠肺部组织EZH2表达明显降低，表明我们的实验成功建立肺部*EZH2*基因敲除的小鼠模型。

肺癌由于受环境、基因等多因素的影响，其发病机制和影响因素都较为复杂，通过构建肺部基因敲除的动物模型，可以更方便有效地认识肿瘤发生发展规律，研究肺癌相关的防治措施并加以评价。通过特异性敲除疾病相关的目的基因，引起特定的蛋白或细胞因子缺失来构建出自发性动物疾病模型现在已得到越来越多人的认同。EZH2作为表观遗传学研究中一个重要的组蛋白甲基转移酶与肿瘤的发展存在密切关系，其表达与组织学分化程度、肿瘤大小、临床分期、淋巴结转移和预后密切相关^[27]^。深入研究EZH2，将有利于探讨其在肿瘤发生、发展中发挥的作用。为了研究特定基因*EZH2*在机体内的具体功能及作用机制，需要抑制该基因表达或敲除目的基因，直接敲除目的基因与利用外界化学或生物条件抑制基因表达相比，其效果更加明确具体，更能直观地反应基因的作用。本研究应用CRISPR/Cas9基因敲除技术构建肺内基因敲除的小鼠模型，为进一步在整体动物水平研究*EZH2*基因在肺癌发生发展过程中的作用提供了理论依据和实验证据。
